# New Frontiers in the Intrarenal Renin-Angiotensin System: A Critical Review of Classical and New Paradigms

**DOI:** 10.3389/fendo.2013.00166

**Published:** 2013-11-11

**Authors:** Jia L. Zhuo, Fernanda M. Ferrao, Yun Zheng, Xiao C. Li

**Affiliations:** ^1^Laboratory of Receptor and Signal Transduction, Department of Pharmacology and Toxicology, University of Mississippi Medical Center, Jackson, MS, USA; ^2^Department of Medicine, Division of Nephrology, University of Mississippi Medical Center, Jackson, MS, USA

**Keywords:** angiotensin 1-converting enzyme, ACE2, angiotensin II receptor, blood pressure, hypertension, kidney, proximal tubule, signal transduction

## Abstract

The renin-angiotensin system (RAS) is well-recognized as one of the oldest and most important regulators of arterial blood pressure, cardiovascular, and renal function. New frontiers have recently emerged in the RAS research well beyond its classic paradigm as a potent vasoconstrictor, an aldosterone release stimulator, or a sodium-retaining hormone. First, two new members of the RAS have been uncovered, which include the renin/(Pro)renin receptor (PRR) and angiotensin-converting enzyme 2 (ACE2). Recent studies suggest that prorenin may act on the PRR independent of the classical ACE/ANG II/AT_1_ receptor axis, whereas ACE2 may degrade ANG II to generate ANG (1–7), which activates the Mas receptor. Second, there is increasing evidence that ANG II may function as an intracellular peptide to activate intracellular and/or nuclear receptors. Third, currently there is a debate on the relative contribution of systemic versus intrarenal RAS to the physiological regulation of blood pressure and the development of hypertension. The objectives of this article are to review and discuss the new insights and perspectives derived from recent studies using novel transgenic mice that either overexpress or are deficient of one key enzyme, ANG peptide, or receptor of the RAS. This information may help us better understand how ANG II acts, both independently or through interactions with other members of the system, to regulate the kidney function and blood pressure in health and disease.

## Introduction

**Figure 1:**
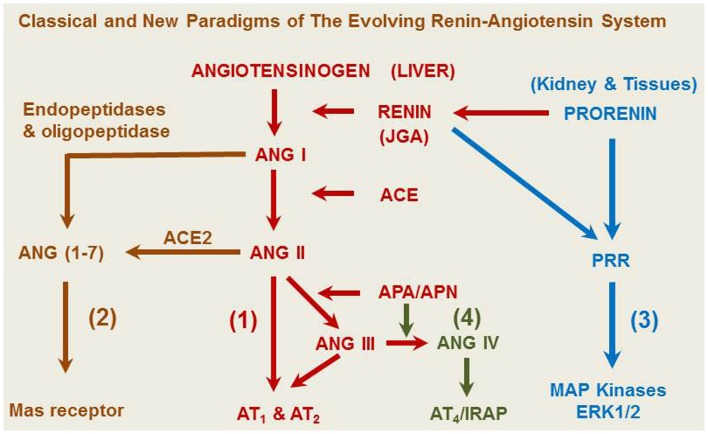
**A representative overview of the evolving renin-angiotensin system**. (1) The classical angiotensinogen/renin/ACE/ANG II/AT_1_ and AT_2_ receptor axis. (2) The prorenin/PRR/MAP kinases ERK 1/2 axis. (3) The ACE2/ANG (1–7)/Mas receptor axis. (4) The ANG IV/AT_4_/IRAP axis. ANG A, angiotensin A. ANG I, angiotensin I. ANG (1–7), angiotensin (1–7). ACE, angiotensin-converting enzyme. ACE2, angiotensin-converting enzyme 2. ANG II, angiotensin II. ANG III, angiotensin III. ANG IV, angiotensin (3–8). APA, aminopeptidase A; APN, aminopeptidase N; AT_1_, type 1 ANG II receptor; AT_2_, type 2 ANG II receptor; IRAP, insulin-regulated aminopeptidase or AT_4_ receptor; JGA, juxtaglomerular apparatus.

Although Tigerstedt and Bergman discovered the rate-limiting enzyme renin about 115 years ago (1), the renin-angiotensin system (RAS) remains to be a remarkable subject for continuous research. Our current understanding of the RAS has greatly evolved from the classical renin/angiotensin-converting enzyme (ACE)/angiotensin II (ANG II)/AT_1_ receptor axis and its physiological roles in the regulation of cardiovascular and renal function, blood pressure, aldosterone biosynthesis and release, and body salt and fluid balance (2–14). However, new frontiers are continuously emerging from the RAS research in recent years, especially in uncovering new enzyme(s) and/or receptor(s) of the system, studying their novel roles, and elucidating their signaling transduction mechanisms. It is now recognized that the classical renin/ACE/ANG II/AT_1_ and AT_2_ axis is no longer the exclusive effector and signaling pathway for the system (15). Three new axes have been recently described to include the ACE2/ANG (1–7)/Mas receptor axis, the prorenin/PRR/MAP kinases ERK1/2 axis, and the ANG IV/AT_4_/IRAP (insulin-regulated aminopeptidase, IRAP) axis (Figure 1) (8, 12, 15–17). The notion that ANG II is the only active peptide of the RAS appears to be outdated, since ANG II can be hydrolyzed by various angiotensinases, ACE2, and neprilysin to generate ANG (1–7), ANG III, ANG IV, and ANG A (2, 16, 18). Prorenin and smaller ANG fragments, including ANG (1–7), ANG III, and ANG IV, can bind their respective receptors or act as an agonist for ANG II receptors to induce a physiological effect (2, 8, 17, 19–21). Indeed, in addition to AT_1_ and AT_2_ receptors that mediate the well-recognized effects of ANG II in the kidney and other tissues, new receptors for prorenin (PRR), ANG (1–7) (Mas receptor), and ANG IV (AT_4_ receptor) have been identified (21–23). Depending on the receptor activated, small ANG peptides may act as an agonist or an antagonist of ANG II. For example, appropriate concentrations of ANG (1–7), ANG III, and ANG IV may activate their respective Mas receptors (8, 9, 16), AT_2_ receptors (19, 24, 25), or AT_4_ receptors to oppose the known effects of ANG II (26, 27). Conversely, high concentrations of ANG (1–7), ANG III, and ANG IV may activate AT_1_ receptors to induce the well-recognized effects of ANG II (16, 20, 28–30). Furthermore, the renin/prorenin receptor, PRR, not only catalyzes prorenin to generate ANG II, but also induces intracellular responses in an ANG II-independent manner (13, 31, 32). Finally, the RAS is no longer considered to act only as an endocrine system, but also acts as a paracrine, autacrine, and intracrine system (33–37). It is likely that ANG II and its smaller ANG peptides may act as both endocrine, paracrine, and intracrine peptides by stimulating cell surface, cytoplasmic and nuclear receptors to exert biological, physiological, and nuclear effects.

The major objective of this article is to review recent advances in biomedical research with a focus on the intrarenal RAS and its paracrine, autacrine, and intracrine roles. New insights, controversies, and perspectives will be discussed by reviewing recent *in vitro* and *in vivo* studies using innovative approaches or animal models including global and tissue-specific RAS transgenic animals. The review article will cover the classical ACE/ANG II/AT_1_ and AT_2_ receptor axis, the ACE2/ANG (1–7)/Mas receptor axis, the prorenin/PRR/MAP kinases ERK1/2 axis, and the ANG IV/AT_4_/IRAP axis. It is expected that this new information may further improve our understanding of physiological and pathophysiological roles of the RAS and help the development of new drugs or strategies to treat hypertension, diabetes, and cardiovascular and kidney diseases by targeting ANG II and other ANG peptides and/or their receptors.

## Current Insights and Future Perspectives on the Roles of the Classical ACE/ANG II/AT_1_ and AT_2_ Receptor Axis in the Kidney

**Figure 2:**
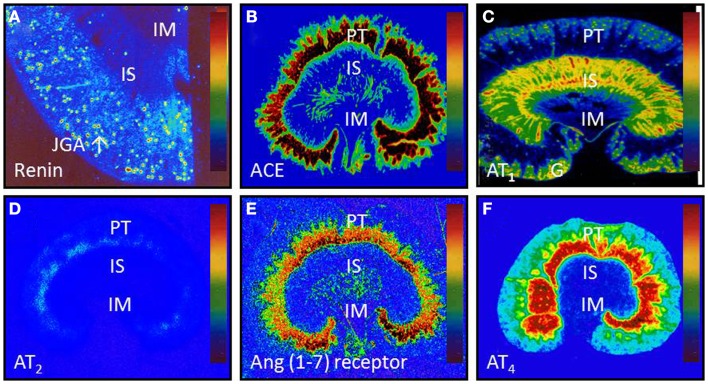
**Intrarenal localization or expression of major components of the renin-angiotensin system**. **(A)** Active renin binding in juxtaglomerular apparatus in the dog kidney using the radiolabeled renin inhibitor, ^125^I-H77. **(B)** ACE binding in the proximal tubule of the rat kidney using ^125^I-351A **(C)** AT_1_ receptor binding in the rat kidney in the presence of the AT_2_ receptor blocker PD123319. **(D)** AT_2_ receptor binding in the rat kidney in the presence of the AT_1_ receptor blocker losartan using ^125^I-[Sar^1^,Ile^8^]-Ang II. **(E)** Ang (1–7) receptor binding in the rat kidney using ^125^I-Ang (1–7) as the radioligand. And **(F)** Ang IV receptor binding in the rat kidney using ^125^I-Ang (3–8). The levels of binding are indicated by color calibration bars with red representing the highest, whereas blue showing the lowest levels of enzyme or receptor binding. G, glomerulus; IM, inner medulla; IS, inner stripe of the outer medulla; JGA, juxtaglomerular apparatus; P, proximal tubule. Reproduced from Li and Zhuo with permission (45).

It is well established that the ACE/ANG II/AT_1_ and AT_2_ receptor axis may function as a circulating or endocrine and paracrine system to regulate cardiovascular, neural, adrenal, and renal function, contributing to normal blood pressure homeostasis and the development of hypertension. However, the specific role of and the extent to which the intrarenal ACE/ANG II/AT_1_ and AT_2_ receptor axis versus the systemic counterpart plays in normal blood pressure control and the development of hypertension remain an issue of continuous debate (10, 38–42). Now, there is a general consensus that all major components of the RAS necessary for generation of ANG II are expressed or present in the kidney (Figure 2) (2, 18, 43–45), and that the levels of ANG II in the kidney are much higher than in plasma (2, 44, 46–49). This is especially true that high ANG II levels have been demonstrated in interstitial and proximal tubular fluid of the kidney and intracellular endosomal compartment (46–48, 50–52).

The mechanisms underlying high levels of ANG II in the kidney are not well understood. In addition to the well-documented expression of all major components of the RAS in the kidney, two major mechanisms may play a critical role under physiological conditions and during the development of ANG II-dependent hypertension. The first is that AT_1_ receptors are abundantly expressed in the kidney, where AT_1_ (AT_1a_) receptor mediates the intracellular accumulation of ANG II especially in proximal tubules (48, 53–58). Classically, a receptor pharmacological dogma suggests that the purpose of G protein-coupled receptor (GPCR)-mediated internalization or endocytosis of an agonist or ligand is to desensitize the cellular responses to the agonist stimulation by moving the agonist/ligand into the cell for degradation in the lysosomal compartment (59–64). The receptor recycles back to the cell membrane to initiate a new round of biological response. However, we and others infused ANG II into rats and mice for 2 weeks, and found no desensitization of ANG II responses, because blood pressure continued to increase and hypertension persists as long as ANG II is infused (48, 53–58). Zhuo et al. reported that in ANG II-infused hypertensive rats, ANG II levels were about 10 times higher in renal cortical endosomes than in control rats via an AT_1_ receptor-mediated mechanism (48). Nishiyama et al. showed that renal interstitial fluid ANG II levels were substantially increased in ANG II-infused rats, an effect also mediated by AT_1_ receptors (65). In AT_1a_ receptor-deficient mice (Agtr1a^−/−^), we further demonstrated that AT_1_ receptor-mediated increases in ANG II uptake in the kidney were largely abolished (57, 58). These studies suggest that AT_1_ (AT_1a_) receptor-mediated uptake of ANG II at least partly contributes to the demonstrated high levels of ANG II in the kidney.

The second classical dogma in the RAS field is that the expression and activity of the RAS is strictly regulated by a negative feedback mechanism by ANG II itself. An increase in the circulating and tissue ANG II is expected to suppress renin release from JGA cells and therefore the production of ANG II in the kidney. However, there is evidence that a positive feed-forward loop exists in the kidney during ANG II-dependent hypertension (43, 44, 66–69). Navar’s group has shown that prorenin and renin (68–70), angiotensinogen (43, 67), and ACE (66) are significantly augmented in response to long-term infusion of ANG II to induce hypertension in rats or mice. Renin and prorenin expression in the collecting ducts are also stimulated during ANG II infusion, likely contributing to increased urinary levels in ANG II-infused hypertensive rats (69–72). Taken together, these studies suggest that in ANG II-infused hypertensive animals, intrarenal ANG II production may be augmented due to increased expression of prorenin and renin, AGT, and ACE.

Currently, there is a great debate on whether AGT, ACE, and AT_1_ receptors in the kidney contribute to the normal blood pressure regulation and the development of hypertension (4, 10, 39–42, 73–77). The classical dogma is that the circulating RAS via the kidney derived renin, liver-derived AGT and vascular endothelial ACE, rather than the intrarenal RAS, plays an important role in the normal blood pressure control and the development of hypertension (78–82). To determine the roles of systemic/endothelial ACE versus tissue/kidney ACE in normal blood pressure and renal control, Bernstein’s group first used targeted homologous recombination to create mice, ACE 2/2, expressing a form of ACE that lacks the COOH-terminal half of ACE with normal or elevated circulating ACE without tissue-bound/kidney ACE (78). Homologous ACE 2/2 mice have significantly lower blood pressure, renal vascular thickening, urine concentrating defect, and significant increase in fractional proximal tubular reabsorption (78). These studies suggests that tissue-bound ACE, rather than circulating ACE, is important for maintaining normal blood pressure (78), and that ACE in the proximal tubule may not be necessary for maintaining normal proximal fluid reabsorption (80). The same group of investigators later generated the so-called ACE 3/3 mice, which is deficient of endothelial ACE in the lung, aorta, or any vascular structure (79). ACE activity in the kidney is about 14% that of wild-type mice, but hepatic ACE expression in ACE 3/3 mice is almost 90-fold that of wild-type. Interestingly, basal blood pressure, plasma ANG II levels, response to ACE inhibitors, and renal function of ACE 3/3 mice were similar to those of wild-type mice. The underlying conclusion of this study is that endothelial ACE is not required for maintaining normal blood pressure and renal function (79). Sen’s group also generated two different strains of mutant mice that express ACE either in vascular endothelial cells (Ts strain) or in renal proximal tubules (Gs strain) (81, 82). Both mutant mice show equivalent serum ACE and ANG II levels, normal kidney structure and fluid homeostasis. In contrast to Bernstein’s ACE3/3 mice (79), only those mutant mice that expressed ACE in vascular endothelial cells had normal blood pressure (81). Proximal fluid reabsorption was found to be normal in the chronic absence of proximal tubule ACE (82). Thus there is still a lack of consensus with respect to the precise roles of systemic/endothelial versus tissue/kidney ACE in normal blood pressure control.

Recently, Gonzalez-Villalobos et al. further determine the role of intrarenal ACE in the normal blood pressure regulation and the development of ANG II-induced hypertension (10, 75). First, Gonzalez-Villalobos et al. also used targeted homologous recombination to generate mice, ACE9/9, that express ACE only in the kidney tubules but not in other tissues (75), or mice with complete deficiency of the entire kidney ACE, ACE 10/10 (10). Similar to Sen’s Gs strain (82), ACE 9/9 mice had lower blood pressure, associated with reduced circulating ANG II, but maintained normal kidney ANG II levels. ACE 9/9 mice responded to chronic ANG I infusion to substantially increase blood pressure (75). In ACE 10/10 mice whose basal blood pressure was similar to wild-type mice, the blood pressure responses to 2-week of ANG II infusion were substantially attenuated in the kidney ACE-KO mice (10). The later study indicates that intrarenal ACE plays a key role in the development of ANG II-induced hypertension, whereas the absence of ACE in the kidney protects against hypertension (10).

However, a careful evaluation of these studies on different strains of ACE mutant mice evokes more questions than answers in the current debate on the relative roles of circulating and intrarenal ACE and therefore ANG II in the blood pressure regulation and the development of hypertension (39, 83). For example, mice with the lack of vascular endothelial ACE may be normotensive (79) or hypotensive (75, 81). Conversely, mice with the lack of kidney/proximal tubular ACE may be normotensive (10, 81). ACE/ANG II appear not to be necessary for maintaining normal proximal tubular fluid reabsorption in mice with overexpression or deficiency of ACE in the proximal tubule (79–82) or the entire kidney (10). Furthermore, circulating or kidney ANG II levels may be normal in these ACE transgenic mice despite of the lack of systemic/endothelial or kidney/proximal tubular ACE (10, 75, 79, 82). These contradictory biochemical, blood pressure, and proximal tubular transport phenotypes, as revealed in various mutant ACE-knockout mice, are difficult to reconcile with well-recognized roles of ACE in the formation of ANG II in the circulation and the kidney, in promoting sodium reabsorption in the proximal tubule and other tubular segments, and in maintaining normal blood pressure homeostasis. However, these diverse phenotypes may provide a new insight into an important role of AT_1_ (AT_1a_) receptor-mediated uptake of circulating ANG II by the kidney, especially in the proximal tubule, in maintaining normal levels of ANG II in the kidney of ACE9/9 and/or ACE10/10 mice (10, 75). As discussed previously, AT_1_ (AT_1a_) receptor-mediated uptake of circulating ANG II at least partly contributes to higher basal ANG II levels and increased ANG II levels in the kidney during ANG II-induced hypertension (48, 54, 57, 58, 84, 85). Another new insight derived from these mutant ACE mouse models is that blood pressure and proximal tubule phenotypes of these ACE-knockout mice are likely complicated by the fact that ACE is chiefly responsible for the metabolism of bradykinin, ANG (1–7), and many other vasoactive peptides such as substance P (8, 9, 18, 86). Knockout of systemic and/or kidney ACE would lead to marked decreases in circulating and intrarenal ANG II and generation of other vasodepressor substances in the circulation and kidney, which may alter blood pressure and renal responses to ANG II or other vasoactive substances under physiological as well as pathophysiological conditions.

**Figure 3:**
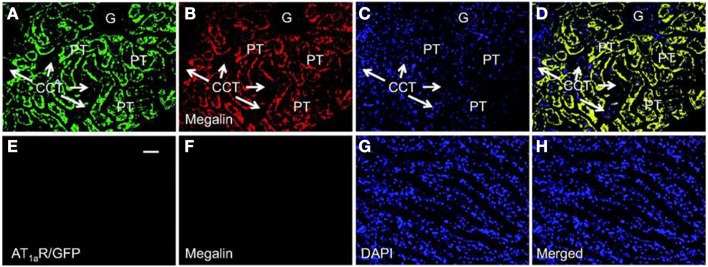
**Proximal tubule-specific expression of AT_1a_R/GFP in a representative Agtr1a^−/−^ mouse kidney 2 week after intrarenal adenoviral transfer**. **(A)** AT_1a_R/GFP expression (green) in proximal tubules (PT). **(B)** Alexa Fluor 594-labeled megalin expression (red) in proximal tubules. **(C)** DAPI-stained nuclei (blue) in the same kidney section. **(D)** Merged image of **(A–C)**, showing the colocalization of AT_1a_R/GFP and megalin expression (yellow) in proximal tubules. Only very low levels of AT_1a_R/GFP and megalin expression are visible in the glomerulus (G) and cortical collecting tubules (CCT). **(E)** AT_1a_R/GFP expression in the outer medulla. **(F)** Alexa Fluor 594-labeled megalin expression in the outer medulla. **(G)** DAPI-stained nuclei in the outer medulla. **(H)** Merged image of **(E–G)**, showing the lack of AT_1a_R/GFP and megalin expression in the outer medulla. Magnification: ×40. Reproduced from Li and Zhuo with permission (42).

**Figure 4:**
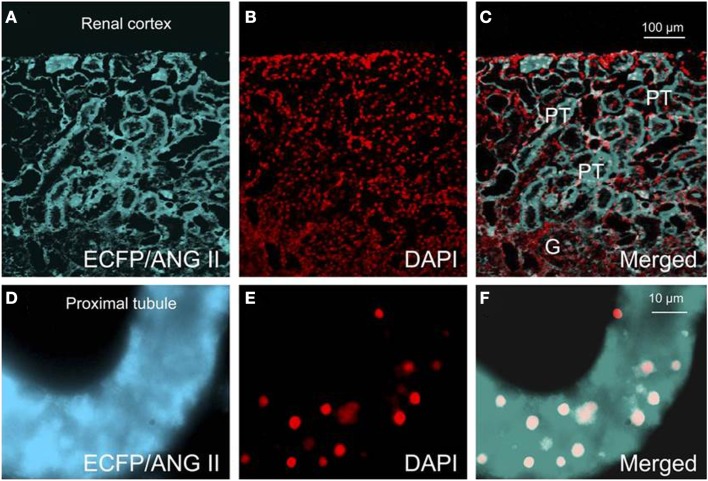
**Effects of proximal tubule-specific, adenovirus-mediated transfer of ECFP/ANG II on ECFP/ANG II expression in the renal outer cortex and freshly isolated proximal tubule of mouse kidneys 2 wk after gene transfer**. **(A)** ECFP expression (blue-green). **(B)** DAPI-stained nuclei (red). **(C)** Merged image of **(A,B)**, respectively, in the outer renal cortex of a representative rat transferred with ECFP/ANG II selectively in proximal tubules. **(D–F)** Expression of ECFP/ANG II in a freshly isolated representative proximal convoluted tubule. Bars = 100 μm for the renal cortex and 10 μm for the isolated proximal tubule. G, glomerulus; PT, proximal tubule. Reproduced from Li et al. with permission (77).

Recent studies using mice with kidney or proximal tubule-specific knockout of AT_1_ receptors provide new insights and perspectives into the roles of the kidney or proximal tubular AT_1a_ receptors in the normal blood pressure regulation and the development of hypertension (4, 38, 40–42, 77, 87). Coffman and Crowley’s group has been instrumental to use the kidney cross-transplantation approach between wild-type and global AT_1a_ receptor-knockout mice (*Agtr1a^−/−^*) (4, 38, 87). These investigators transplanted the kidney of wild-type mice into *Agtr1a^−/−^* mice to generate systemic AT_1a_-KO mice, and conversely transplanted the kidney of *Agtr1a^−/−^* mice into wild-type mice to generate the kidney-specific AT_1a_-KO mice. Blood pressure and cardiac hypertrophic responses to ANG II infusion or high salt intake were compared in the systemic- and kidney-specific AT_1a_-KO mice (4, 38, 87). These elegant studies confirmed that the kidney AT_1_ receptors are absolutely required for the development of ANG II-dependent hypertension and cardiac hypertrophy, and systemic AT_1_ receptors is not sufficient for ANG II to induce hypertension or cardiac hypertrophy (38). Using the Cre/Lox strategy, Gurley et al. (40) and Li et al. (41) generated proximal tubule-specific AT_1a_-KO mice to determine the role of proximal tubule AT_1a_ receptors in blood pressure regulation. Both studies demonstrated that deletion of AT_1a_ receptor and its signaling in the proximal tubule alone is sufficient to significantly decrease basal blood pressure, despite intact systemic AT_1a_ receptor expression and vascular responses (40, 41). Alternatively, we have recently produced adenoviral constructs encoding GFP-tagged AT_1a_ receptor gene (AT_1a_R/GFP) (Figure 3), or an enhanced cyan fluorescent protein (ECFP)-tagged ANG II fusion protein, and a proximal tubule-specific sodium and glucose cotransporter 2 (sglt2) promoter (Figure 4) (42). We demonstrated that intrarenal transfer of AT_1a_R/GFP alone selectively in the proximal tubule was sufficient to increase systolic blood pressure by ∼12 mmHg 14 days after the gene transfer (42). Cotransfer of AT_1a_R/GFP with ECFP/ANG II increased blood pressure further to 18 mmHg. The increases in blood pressure were associated with twofold increases in phosphorylated MAP kinases ERK1/2, lysate and membrane NHE3 proteins in freshly isolated proximal tubules, and a decrease in 24 h urinary sodium excretion (42). Taken together, these elegant studies strongly suggest that the proximal tubule ACE/ANG II/AT_1a_ receptor axis via promoting proximal tubular sodium and fluid reabsorption may contribute approximately 15 mmHg to basal blood pressure homeostasis in mice.

## Current Insights and Future Perspectives on the Roles of the ACE2/ANG (1–7)/Mas Receptor Axis in the Kidney

ANG (1–7) is the most extensively studied smaller ANG peptide in the RAS since 1970s (8, 9, 17, 18, 88). Early studies showed that structural deletion of either phenylalanine (position 8) or the dipeptide, Pro-Phe (positions 7 and 8) from ANG II completely removed the vasoconstrictor, central pressor, or thirst-stimulating actions of ANG II (89). The structural and activity studies suggested that ANG (1–7) may be an inactive component of the RAS. However, subsequent studies primarily from Ferrario’s group demonstrated that ANG (1–7) has significant vasodepressor and antihypertensive actions in hypertensive animals or humans, which may oppose the actions of ANG II either directly or indirectly by stimulation of prostaglandins and nitric oxide (8, 9, 17, 18, 88). The importance of this heptapeptide in cardiovascular, blood pressure, and renal control gains further recognition recently upon the molecular characterization of a GPCR using ANG (1–7) as a ligand, the Mas receptor (23). It is increasingly recognized that the new ACE2/ANG (1–7)/Mas receptor axis acts to counteract most of the known deleterious actions of the ACE/ANG II/AT_1_ receptor axis (8, 16, 17). However, recent studies on transgenic animals overexpressing ANG (1–7) have provided new insights and perspectives on whether ANG (1–7) plays beneficial cardiovascular, blood pressure, and renal hemodynamic effects (90–92).

The kidney is one of the key tissues in which ANG (1–7) is generated from the metabolism of ANG II by ACE2 with the proximal tubule exhibiting the most robust ACE2 activities (8, 49). ANG (1–7) can be easily detected in the proximal tubule and urine of rats, sheep, and humans, but it can be rapidly hydrolyzed to ANG (1–5) and ANG (1–4) by ACE and neprilysin (8, 49). Whether ANG (1–7) is primarily produced from the degradation of ANG II by ACE2 in the circulation and kidney remains an issue of continuous debate. An early study by Yamamoto et al. showed that infusion of ANG II in WKY or SHR rats was not accompanied by significantly increased plasma ANG (1–7) levels (93). Modrall et al. reported that in tissue ACE-knockout mice, intrarenal ANG I and ANG II levels were decreased by 70–80% compared with wild-type mice, but ANG (1–7) levels were surprisingly normal in the kidney (94). Thus a more balanced view may be that ANG (1–7) is derived from both the metabolism of ANG I via the endopeptidase-dependent pathway and the metabolism of ANG II by the ACE2-dependent pathway.

Both renal hemodynamic and tubular effects have been demonstrated although the signaling mechanisms involved are not fully understood (17). However, the current insight is that ANG (1–7) acts primarily to oppose the cardiovascular and renal effects of ANG II. For example, ANG II is known to increase blood pressure, induce renal vasoconstriction to decrease renal blood flow (RBF) and glomerular filtration rate (GFR), and induce antidiuresis and antinatriuresis (43, 95–98). By contrast, ANG (1–7) infusion generally opposes and attenuates these effects of ANG II (8, 16, 17, 36, 99). The diuretic/natriuretic effects of ANG (1–7) may be partly due to the renal vasodilatation as well as inhibition of sodium and water reabsorption along the nephron segments. Previous studies demonstrated that ANG (1–7) may be a potent inhibitor of Na^+^-K^+^-ATPase in the proximal tubule (16, 17). ANG (1–7) may inhibit Na^+^-K^+^-ATPase via AT_2_ receptor-mediated stimulation of the G(i/o) protein/cGMP/PKG signaling pathway (100, 101). Moreover, ANG (1–7) showed biphasic effects on the Na^+^/H^+^ exchanger activity in isolated proximal tubules mediated by the Mas receptor and changes in [Ca^2+^]_i_ (30, 102). In rat inner medullary collecting ducts (IMCD), ANG (1–7) enhanced water transport via the vasopressin V_2_ receptor (103). However, some of renal effects induced by ANG (1–7) are very difficult to reconcile with the dogma on the potential roles of the ACE2/ANG (1–7)/Mas receptor axis to counteract with detrimental roles of the renin/ACE/ANG II/AT_1_ receptor axis. A careful review of the above-mentioned studies reveals that ANG (1–7) may also activate the well-recognized downstream ANG II/AT_1_ receptor signaling transduction to induce similar effects induced by ANG II.

New insights and perspectives into the physiological roles of ANG (1–7) acting via the Mas receptors in the cardiovascular, blood pressure, and renal regulation may be best inferred from transgenic animals with overexpression of ANG (1–7) (90, 91, 104) or ACE2 (105–107) to substantially increasing production of ANG (1–7) in the circulation or tissues or due to global or tissue-specific deletion of the Mas receptor. Santos’ group has generated transgenic rats that express an ANG (1–7)-producing fusion protein, TGR(A1–7)3292, in the testis (90). Expression of ANG (1–7) in the testis acts as an ANG (1–7) biological pump to increase the plasma ANG (1–7) concentration 2.5-fold. Surprisingly, overexpression of ANG (1–7) did not alter basal blood pressure levels in TGR(A1–7)3292 rats despite of significant increases in stroke volume and cardiac index and a decrease in total peripheral resistance (90, 104). While acute intravenous infusion of ANG (1–7) induces renal vasodilatation, diuresis, and natriuresis (17, 99), GFR and 24 h urinary sodium excretion in TGR(A1–7)3292 rats are similar to those in Sprague-Dawley rats, whereas 24 h urine excretion was decreased and osmolality increased, respectively (91). The results obtained from TGR(A1–7)3292 rats appear to be contradictory to the well-known vasodepressor, diuretic and natriuretic effects of ANG (1–7). In a different study, Rentzsch et al. generated transgenic rats on a SHRSP genetic background expressing the human ACE2 in vascular smooth muscle cells by the use of the SM22 promoter, SHRSP-ACE2 (105). SHRSP-ACE2 rats have significantly elevated circulating levels of ANG (1–7), which is associated with a 15 mmHg decrease in mean arterial blood pressure and significantly attenuated responses to ANG II (105). These data suggest that vascular ACE2 overexpression may be a novel therapeutic strategy in the treatment of hypertension. Liu et al. used the adenoviral gene delivery approach to overexpress ACE2 globally and found that blood pressure was not different between control and ACE2-overexpressing Wistar rats before and after streptozotocin treatment to induce diabetic nephropathy (106). Despite of these inconsistencies, global or tissue-specific overexpression of ACE2 has been reported to reduce blood pressure or hypertension-induced injury in SHR (108, 109), and protect from ischemia-induced cardiac injury (110), and attenuate diabetic nephropathy (106).

Although the GPCR Mas was reported to be the specific receptor for ANG (1–7) more than 10 years ago (23), there is surprisingly little progress that has been made in using these Mas receptor-deficient mice (Mas-KO) to determine the physiological roles of ANG (1–7) (111–114). Too often, the reported cardiovascular, blood pressure, and renal phenotypes are sometimes contradictory between studies. Botelho-Santos reported that mean arterial pressure in anesthetized Mas-KO mice (12–16 weeks old) was not different from that of WT mice, despite of significant decreases in stroke volume and cardiac index and marked increases in vascular resistance and a decrease in blood flow in the kidney (115). Walther et al. also confirmed that neither heart rate nor blood pressure was significantly different between Mas-KO mice and controls, although salt-induced increase in blood pressure was prevented in Mas-KO mice (116, 117). Subsequent studies from the same groups of investigators showed a significantly higher basal blood pressure in Mas-KO mice (112, 118). These differences may be explained by the difference in genetic backgrounds, in that the former Mas-KO mice were generated from mixed genetic background, 129 × C57BL/6, whereas the latter were generated from the FVB/N genetic background for seven generations (16, 119). Other studies supporting the counterregulatory roles of the ACE2/ANG (1–7)/Mas receptor axis against those of the ACE/ANG II/AT_1_ receptor axis in the kidney include the development of glomerular hyperfiltration and microalbuminuria in Mas-KO mice (120). However, Esteban et al. recently shown that ANG (1–7), via the Mas receptor, has proinflammatory properties at least as potent as those of ANG II and TNFα in the kidney (121). Clearly, controversies remain with respect to the specific roles of the Mas receptor in mediating the effects of ANG (1–7) in the kidney (122).

## Current Insights and Future Perspectives on the Roles of the Prorenin/PRR/MAP Kinases ERK 1/2 Axis in the Kidney

A new frontier in the RAS research field emerges during recent years is the prorenin/PRR/MAP kinases ERK 1/2 axis. According to the classical dogma, prorenin is primarily synthesized in the juxtaglomerular (JGA) cells and is biologically inactive (123). Prorenin becomes active renin in JGA cells and is released in response to a decrease in blood pressure (hypotension), activation of renal sympathetic nerves, and sodium depletion. Renin released from JGA cells initiates the activation of the RAS by hydrolyzing circulating and tissue AGT to generate ANG I (123). This classical dogma may be subject to significant revisions as a result of recent progresses being made in the field.

There is strong evidence that prorenin may also be constitutively secreted from the kidney, and to a less extent from extrarenal tissues including eyes and adrenal glands (11–13, 22, 124–126). Whether prorenin is physiologically or pathophysiologically relevant remains an issue of intensive debate before and after Ngyuen et al. first cloned the prorenin/renin receptor (PRR) (22, 127). PRR has a single transmembrane domain and 350-amino acid (22, 127). It has specific binding site not only for the inactive precursor prorenin, but also for active renin, which is the key initiator of the ACE/ANG II/AT_1_ receptor axis. Thus it is difficult to determine whether it is prorenin or active renin that binds and activates PRR under physiological conditions and in cardiovascular, diabetic and renal diseases. However, it has been shown that prorenin has a “handle” region with higher affinity for PRR than renin, which binds to PRR to initiate the catalytic activity of prorenin, leading the activation of the prorenin/PRR/MAP kinases ERK1/2 axis (12, 22, 127). It has been further suggested that a decoy “handle” region peptide (HRP) may thus target this “handle” region by competitively inhibiting the binding of prorenin to the PRR, and produce pharmacological and therapeutical effects in treating cardiovascular, hypertensive, and diabetic diseases (31, 128, 129). Whether HRP may specifically block PRR to exert beneficial therapeutic effects remains highly controversial (13, 126, 130). Several studies have been unable to confirm the role(s) of prorenin and the effects of HRP in cultured cells and animals (131–133). Even if HRP is indeed effective in blocking prorenin and PRR interactions, its clinical relevance remains unknown due to its peptide properties. The renin-specific inhibitors have been developed to treat hypertension and cardiovascular and kidney diseases. Whether the renin inhibitors are therapeutically superior to classical ACE inhibitors or ARBs remains to be determined. If prorenin and PRR indeed play important physiological and pathophysiological roles in blood pressure regulation and pathologies of cardiovascular, renal, and diabetic diseases, the development of orally active PRR-specific inhibitors to block prorenin-induced activation of PRR will be highly necessary.

While prorenin and renin are present primarily in JGAs of the renal cortex under physiological conditions, PRR is reportedly expressed in glomerular mesangial cells and the subendothelium of renal arteries (22), and in the apical membrane of intercalated cells in collecting ducts (134). Activation of PRR by the rat recombinant prorenin has been shown to stimulate cyclooxygenase-2 (COX-2)-derived prostaglandins via MAP kinases 1/2 in rat renal inner medullary collecting duct cells (IMCD) (135). Furthermore, prorenin appears to activate the prorenin/PRR/MAP kinases ERK 1/2 axis to increase V-ATPase activity (vacuolar-type H^+^-ATPase) at nanomolar concentrations in intercalated cells, MDCK.C11 (136). PRR has been described as an accessory subunit for V-ATPase, and may function as a H^+^-ATPase subunit in distal nephron segments of the kidney (137). However, Oshima et al. reported that PRR may be necessary for the maintenance of normal podocyte structure and function (138).

Activation of PRR by prorenin may be implicated in the development and progression of renal diseases in animal models. Kaneshiro et al. generated transgenic rats with overexpression of human prorenin/renin, and showed that these rats slowly developed nephropathy via MAP Kinases ERK1/2 signaling through an ANG II-independent mechanism (139). Ichihara et al. showed that the prorenin/PRR/MAP kinases ERK1/2 axis plays a pivotal role in the development of diabetic nephropathy in ANG II AT_1a_ receptor-deficient mice (129) and in diabetic rats (128). Furthermore, Prieto and Navar’ group has shown that prorenin and PRR expression are markedly increased in the collecting ducts of distal nephron in ANG II-induced and 2K1C renal hypertension, although the precise roles of prorenin and PRR as a byproduct or mediator of ANG II-dependent hypertension remain unknown (69, 72).

Overall, prorenin and PRR have been studied extensively during last several years and appear to play important roles under certain biological, physiological, and pathophysiological conditions or animal models (12, 140, 141). However, their specific roles in the physiological regulation of cardiovascular, blood pressure, and renal function and the development of cardiovascular, hypertensive, and renal diseases in humans remain to be confirmed (13, 126). Recently, Reudelhuber (13) and Campbell (126) have provided excellent critical reviews in these issues. One key issue is that mice is known to express abundant prorenin and PRR than rats and humans, but they do not develop hypertension or cardiovascular and renal diseases. Another issue is that it is difficult to prove the activation of PRR by prorenin independent from renin without genetic deletion of PRR in mice, which is lethal at present (142, 143). The third issue is that prorenin may be overexpressed in transgenic rats or mice with hundreds or even thousands of time higher than those in humans to manifest cardiovascular, blood pressure, and renal phenotypes, which is unlikely replicated in normal and diseased humans (125, 144, 145). Finally, some, if not all, prorenin-induced blood pressure and cardiovascular and renal responses remain to be ANG II/AT_1_ receptor-dependent (13, 32, 126).

## Current Insights and Future Perspectives on the Roles of Intracrine or Intracellular ANG II in the Kidney

A new frontier in the RAS research field has recently gained increasing attention (33–37, 146). This is now recognized as an intracrine or intracellular RAS. Many tissues or cells may synthesize ANG II within the cells, wherein ANG II bind to intracellular and/or nuclear receptors, activate downstream signaling pathways, and induce cellular and/or nuclear responses independent of cell surface receptors (33, 147–150). Alternatively, we and others have shown that circulating, paracrine, and autacrine ANG II may enter cells via AT_1_ (AT_1a_) receptor-mediated uptake or internalization in the kidney, primarily in the proximal tubule (48, 52, 57, 58, 151, 152). There is substantial evidence that not all internalized ANG II are degraded in lysosomes as the classical receptor pharmacology dogma suggests, and ANG II may escape from degradation by lysosomes. For example, systemically infused ^125^I-labeled ANG I or ^125^I-ANG II have been identified and quantified in pig kidney cells (55, 56, 85) and rat kidney cells (153, 154). Imig et al. demonstrated ACE, ANG II and AT_1a_ receptors in cortical endosomes of the rat kidney (52). In ANG II-infused rat kidney, we found that ANG II levels in the renal cortical light and heavy endosomes were up to 10-fold higher compared with control rats (48). Intracellular accumulation of ANG II in the proximal tubule of the kidney may be blocked by the AT_1_ receptor blockers candesartan (48), losartan or in AT_1a_-KO mice (57, 58). To further support the new intracellular ANG II paradigm, specific and functional AT_1_ (AT_1a_) and AT_2_ receptors have been demonstrated in rat renal cortical endosomes (48, 52), mouse kidney proximal tubule mitochondria (155), and rat or sheep renal cortical nuclei (156–159). Thus the localization of intracellular and/or nuclear ANG II and AT_1_/AT_2_ receptors provides evidence that ANG II may interact with AT_1_/AT_2_ receptors within the kidney cells to induce biological and physiological effects.

In the kidney, previous studies demonstrated that AT_1a_ receptor-mediated endocytosis of ANG II is required for ANG II-stimulated proximal tubular sodium transport or uptake of ^22^Na^+^ (160–163). We also showed that AT_1a_ receptor-mediated ANG II uptake was associated the inhibition of cAMP signaling (151), activation of NF-κB signaling (163), and increases in lysate and membrane phosphorylated NHE3 proteins in proximal tubule cells (164). However these studies by no means provide direct evidence to support the role of intracellular and/or nuclear ANG II in the regulation of renal function and blood pressure responses. Several recent proof-of-concept studies have provided some new insights and perspectives into the potential roles of intracellular ANG II in the kidney. First, we used the single cell microinjection approach as described by Haller et al. (149) to determine the role of intracellular ANG II and its receptors in mobilizing intracellular calcium responses in rabbit proximal tubule cells (150). While the cell surface AT_1_ receptors were blocked by losartan in the medium, ANG II was directly microinjected into single monolayer proximal tubule cells sub-cultured on glass coverslips with or without the AT_1_ receptor blocker losartan or the AT_2_ receptor blocker PD123319. Microinjection of ANG II evoked marked increases in intracellular calcium responses, which were largely blocked by concurrent microinjection of losartan, but not by PD123319, indicating an AT_1_ receptor-mediated response (150). In a subsequent proof-of-concept study, we isolated fresh nuclei from the renal cortex of the rat kidney and incubated the nuclei with ANG II in an *in vitro* transcriptional system to determine the transcriptional effects of ANG II (156). We demonstrated that ANG II directly stimulated nuclear AT_1a_ receptors to increase *in vitro* transcription of mRNAs for TGF-β1, MCP-1, and NHE3, which are known to play important roles in cell proliferation and hypertrophy, tissue fibrosis, and sodium transport in the kidney. Again, these nuclear transcriptional responses to ANG II were blocked by losartan but not by PD123319, further underlying an important role of AT_1_ (AT_1a_) receptors in proximal tubule cells. In alternative proof-of-concept studies, Chappell’s group showed that ANG II and ANG (1–7) directly stimulated nuclear AT_2_ or ANG (1–7) receptors to increase NO production, and activated AT_1_ receptors to increase super oxide production in freshly isolated sheep kidney nuclei (157, 158, 165).

Although it has been hypothesized that intracellular ANG II may play a physiological role in the cardiovascular and renal systems and blood pressure regulation, there was no direct evidence supporting this role until recently. Cook’s group was instrumental in generating transgenic mice that globally express an ANG II fused downstream of ECFP in all tissues, and its expression was driven by the mouse metallothionein promoter (146). The fusion protein, ECFP/ANG II, lacks a secretory signal, so its expression is retained intracellularly. Although plasma ANG II was not altered in these transgenic mice, basal blood pressure was significantly increased by approximately 16 mmHg, and thrombotic microangiopathy or microthrombosis was developed within the glomerular capillaries and small vessels (146). This study demonstrated for the first time that overexpression of an intracellular ANG II fusion protein is sufficient to elevate basal blood pressure and induce renal pathology. To determine the role of intracellular ANG II in the regulation of proximal tubular reabsorption and blood pressure, we performed intrarenal transfer of the same ECFP/ANG II selectively in the proximal tubule of rats and mice (Figures 3 and 4) (42, 77, 166). We showed that proximal tubule-specific overexpression of intracellular ECFP/ANG II significantly increased blood pressure by approximately 15–20 mmHg in rats and C57BL/6J mice 7 days after the gene transfer, and the blood pressure responses were blocked by losartan treatment or in AT_1a_-KO mice (42, 166, 167). Furthermore, the hypertensive effects of proximal tubule-specific ECFP/ANG II expression were associated with decreases in 24 h urinary sodium excretion, increases in phosphorylated ERK1/2, lysate, and membrane NHE3 proteins in freshly isolated proximal tubules and decrease in fractional lithium excretion (42, 166, 167). These responses are consistently with the concept that intracellular ANG II may stimulate AT_1_ receptor to increase proximal tubular sodium and fluid reabsorption, which in turn contributes to the regulation of blood pressure.

## Current Insights and Future Perspectives on the Roles of ANG III, ANG IV, or ANG A in the Kidney

Two other smaller ANG peptides, ANG III and ANG IV, have been reported to have significant effects on blood pressure and renal function (2, 18, 19, 24, 28, 168). ANG III, ANG (2–8), is derived from the metabolism of ANG II by aminopeptidase A. To date, there is no evidence for a specific ANG III receptor. In the kidney, ANG III normally binds to the AT_1_ receptor and AT_2_ receptors, and the reported natriuretic and antinatriuretic effects of ANG III may be dose-dependent on whether the AT_1_ or AT_2_ receptor is activated (2, 18, 28, 168). When centrally administrated, ANG III appears to enhance vasopressin release, thirst, and blood pressure (169). Most recently, Carey’s group has shown that intrarenal interstitial ANG III infusion induced natriuresis via the AT_2_ receptor/nitric oxide/cGMP-dependent mechanism (19, 24, 170).

In the kidney, ANG III can be further hydrolyzed by aminopeptidase N to generate ANG IV or ANG (3–8) (2, 18, 171, 172). The receptor for ANG IV, AT_4_, has been identified as an IRAP, associated with the M1 family of aminopeptidases and GLUT4 vesicles in insulin-responsive cells (21, 173). The AT_4_ receptor has been localized in different tissues in the brain, heart, blood vessels, and kidney (20, 26, 174, 175). It is worth mentioning that other peptides such as LVV-hemorphin 7 also bind the AT_4_ receptor (21, 175, 176), and ANG IV also stimulates the AT_1_ receptor (20, 177–179). ANG IV is implicated in the regulation of learning and memory in rodents and improves memory in animal models of amnesia, and has been suggested to be used to treat Alzheimer’s disease (21, 175, 176). Aminopeptidases A and N are particularly abundant in the kidney, especially in the glomeruli and proximal nephron segment (2, 18, 171, 172). We have previously shown that nanomolar concentrations of ANG IV may increase blood pressure and induce renal vasoconstriction via the AT_1_ receptor-activated signaling in anesthetized rats (20), but others showed increased renal cortical blood flow and decreases Na^+^ transport in isolated renal proximal tubules (26, 27). Furthermore, Ang IV infusion into the renal artery decreased RBF, without any change in blood pressure, suggesting an AT_1_-mediated constriction in renal vascular bed (180). Other Ang IV responses in different kidney cells appear to occur via AT_1_ receptor activation as well, such as Ca^2+^ mobilization in glomerular mesangial cells (20, 178), and in human proximal tubules cells (181). In wild-type and AT_1a_, AT_1b_, AT_2_ receptor and IRAP knockout mice, Ang IV was found to mediate blood pressure and renal vasoconstrictor effects via AT_1a_ receptors (182, 183). Thus, the physiological roles of ANG IV/AT_4_ receptors in blood pressure and renal regulation remain uncertain, given that circulating and tissue ANG IV levels are unlikely to be higher than those of ANG II in health and disease and that ANG IV also binds and stimulates AT_1_ receptors.

Recently, an ANG peptide-derived fragment called ANG A (Ala-Arg-Val-Tyr-Ile-His-Pro-Phe) has been described in the plasma of healthy humans and with increased concentrations in end-stage renal failure patients (184–186). ANG A may be generated from ANG II by decarboxylation of Asp^1^ and have the same affinity for AT_1_ receptor as ANG II, and higher affinity for AT_2_ receptor (186, 187). In rats, ANG A and ANG II have similar hypertensive effects, but ANG A possesses a greater proliferative effect on vascular smooth muscle cells than ANG II (186, 187). In genetically modified mice and in normotensive and hypertensive rats, ANG A induces pressor and renal vasoconstrictor responses also in the AT_1_ receptor-dependent manner (186). The role(s) of ANG A and its receptor-mediated downstream signaling mechanisms remain incompletely understood. However, since the ANG II/AT_1_ receptor-dependent pathways are involved, the translational impact of the ANG A research may likely be limited because the available ARBs are expected to block the actions of ANG A in tissues.

## Concluding Remarks

In summary, the RAS has evolved from a circulating and endocrine system to multiple endocrine, paracrine, and intracrine systems. At least four axes for the RAS have been identified in the kidney and other tissues (Figure 1) and their physiological and pathophysiological roles explored. These include the most-studied and recognized classical renin/ACE/ANG II/AT_1_ and AT_2_ receptor axis, and three new axes including the ACE2/ANG (1–7)/Mas receptor, the prorenin/PRR/MAP kinases ERK1/2, and the ANG IV/AT_4_/IRAP axis. Each of these axes has its own enzyme(s), substrate(s), agonist(s), or ligand(s), respective receptor and downstream signaling mechanisms. Thus the roles of the RAS have been extended far beyond the regulation of blood pressure, aldosterone synthesis, and body salt and fluid homeostasis by the AT_1_ and AT_2_ receptors. Indeed, novel actions have been described for each axis of the entire RAS, interactions of which undoubtedly contribute to the overall regulation of cardiovascular, neural, and renal function and blood pressure. It is now well understood that imbalance of actions induced by ANG II and its smaller metabolites, ANG (1–7), ANG III, and ANG IV in favoring increases in tissue ANG II formation and the activation of the ACE/ANG II/AT_1_ receptor axis may lead to the development of hypertension and ANG II-induced target organ injury and diseases. Conversely, genetic and pharmacological approaches to increase the production of ANG (1–7) via overexpression of ACE2 or ANG (1–7) fusion protein may partially oppose the well-recognized actions of ANG II through activation of the Mas receptor. However, despite of the great progress new challenges still remain in the RAS research field. For example, the challenges for studying the classical ACE/ANG II/AT_1_ receptor axis may include determining the roles of intracellular and nuclear ANG II and its receptors play in the nuclear and/or transcriptional responses to ANG II in various diseases, and developing novel molecular and pharmacological approaches or drugs to block the transcriptional actions of intracellular ANG II. Since ANG III, ANG IV, and ANG A may also function as potent agonists of the AT_1_ and/or AT_2_ receptor to alter blood pressure and renal function, their physiological and pathophysiological roles remain to be determined. Similarly, the challenges for studying the roles of the prorenin/PRR/MAP kinases ERK1/2 axis is how to better differentiate the ANG II/AT_1_ receptor-dependent and independent effects of prorenin/PRR activation, and whether blockade of prorenin activation provides additional and beneficial effects beyond renin and ACE inhibitors or AT_1_ receptor blockers. Finally, although the ACE/ANG (1–7)/Mas receptor axis may play a counterregulatory role to oppose the effects of the renin/ACE/ANG II/AT_1_ receptor axis, the development and clinical relevance of the orally active agonists or compounds that promote metabolism of ANG II to increase ANG (1–7) production or to activate the Mas receptor still await clinical trials.

## Conflict of Interest Statement

The authors declare that the research was conducted in the absence of any commercial or financial relationships that could be construed as a potential conflict of interest.
